# Physicians’ knowledge and adherence to Centor Criteria for preventing acute rheumatic fever in Jeddah, Saudi Arabia

**DOI:** 10.25122/jml-2024-0397

**Published:** 2025-05

**Authors:** Mohammed Shaikhomer, Jumana Hussain Timraz, Nada Yasser Metwali, Fathima Shamma Bava, Zainab Yusuf, Ruqayyah Ali Ahmed, Husna Irfan Thalib, Syeda Nafeesa Hashim

**Affiliations:** 1 Department of Internal Medicine, Faculty of Medicine, King Abdulaziz University, Jeddah, Saudi Arabia; 2 Department of General Medicine and Surgery, Batterjee Medical College, Jeddah, Saudi Arabia

**Keywords:** Acute rheumatic fever, Centor criteria, antibiotics, streptococcal pharyngitis, ARF: Acute Rheumatic Fever, GAS: Group A Streptococcus, ACP: American College of Physicians, CDC: Centre for Disease Control and Prevention, RADT: Rapid Antigen Detection Test, IDSA: Infectious Diseases Society of America, AHA: American Heart Association, MOH: Ministry of Health

## Abstract

Acute rheumatic fever (ARF) is a delayed autoimmune complication of Group A Streptococcal (GAS) pharyngitis and remains a significant public health concern in regions such as Saudi Arabia. Timely treatment with antibiotics guided by the Centor criteria can prevent ARF, yet adherence to these recommended guidelines remains inconsistent among physicians in developing countries. This study aimed to assess the knowledge and adherence of physicians in Jeddah, Saudi Arabia, to the Centor criteria in managing streptococcal pharyngitis as a strategy to prevent ARF. A cross-sectional study was conducted among 105 physicians across various specialties. A structured questionnaire was used to evaluate their adherence and awareness. Data was analyzed using SPSS version 23.0, with chi-square tests to assess the significance (*P* < 0.05). Only 40% of physicians reported consistent use of the Centor criteria. Female and non-Saudi physicians demonstrated higher rates of adherence. The most compliant were consultants (42.8%), while ENT and family medicine departments demonstrated better adherence to Ministry of Health guidelines. Additionally, physicians with over 15 years of experience demonstrated significantly greater adherence than their less experienced counterparts. Significant gaps were identified in the adherence to the Centor criteria, which were influenced by professional roles and demographic factors. Promoting adherence to national guidelines and standardizing training is crucial for improving ARF prevention in Saudi Arabia.

## INTRODUCTION

Acute rheumatic fever (ARF) is an autoimmune disease resulting from a delayed, nonsuppurative, abnormal immune response to Group A Streptococcus (GAS) infection. It commonly affects children between 5 and 14 years old [1]. The prevalence of the disease has reduced drastically in developed countries over the past 50 years; however, it remains a major cause of morbidity and mortality in developing countries, including Saudi Arabia, which falls under one of the high-prevalence regions [2,3]. The prevalence is known to be more than 40 million worldwide and accounts for more than 300,000 deaths annually. However, the numbers are probably underestimated due to underdiagnosis and lack of global data availability [2].

The prevention of ARF relies heavily upon timely intervention and prompt antibiotic treatment of preceding streptococcal infection [4]. The American College of Physicians (ACP), the Centers for Disease Control and Prevention (CDC), and the American Academy of Family Practice (AAFP) recommend using the Centor criteria, which is a clinical scoring system that aims to guide the decision of prescribing antibiotics in suspected cases of streptococcal pharyngitis and to differentiate between viral and streptococcal pharyngitis clinically [5]. The four-point clinical characteristics of the Centor criteria include the history of fever, absence of cough, exudative tonsillitis, and anterior cervical adenopathy [5,6].

Several guidelines aim to diagnose and treat streptococcal pharyngitis based on the Centor criteria. The ACP, CDC, American Society of Internal Medicine, and the AAFP consider (a) a Centor score of 0 or 1 to be of low risk for streptococcal infection and therefore do not recommend any testing or antibiotics prescription, (b) patients with score of 2 or 3 are recommended to be tested by a rapid antigen detection test (RADT), followed by antibiotic prescription for a positive test, (c) patients with score of 3 or 4 are recommended to be empirically treated with antibiotics. On the other hand, the Infectious Diseases Society of America (IDSA), American Heart Association (AHA), and American Academy of Pediatrics (AAP) recommend confirmation of streptococcal diagnosis with RADT or throat culture, regardless of the Centor score, before prescribing antibiotics [5,7]. IDSA guidelines, however, align with APC guidelines regarding neither testing nor prescribing antibiotics to patients with a score of 0 or 1 [5].

In addition to the original Centor criteria, the Modified Centor Criteria, also known as the McIsaac score (Tables 1 and 2), is an updated scoring tool incorporating age as an additional factor. This tool, therefore, can be used in both children and adults. According to the modified Centor criteria, children between 3 and 14 and adults aged 45 or older receive an additional point. Patients with a score of 3 or more points are prescribed antibiotics based on culture or RADT, while patients with a score of 2 or fewer are recommended to be treated symptomatically without further testing [8]. In line with international recommendations, the Saudi Ministry of Health issued the *Group A Streptococcal Pharyngitis Protocol* in November 2021, which advocates using the Modified Centor Criteria for diagnosis and treatment decisions [9].

**Table 1:** Modified Centor Criteria (McIsaac Score) and its interpretation [9].

Modified Centor Criteria	Scores
Absence of cough	1
Tender cervical lymphadenopathy	1
Fever (>38ºC)	1
Tonsillar exudate	1
Children 3-14	1
Adults 15-44	0
45 years and older	-1

**Table 2:** Interpretation of the Modified Centor Criteria (McIsaac Score) [9]

Total Score	Interpretation
≤ 2 points	No further testing is required; symptomatic treatment
≥ 2 points	Antibiotic treatment based on RADT or culture

Several studies highlight the benefits of Centor criteria or Modified Centor Criteria use by physicians in developed countries; however, there is a lack of data on the use of Centor criteria and physician adherence to these guidelines in developing countries, which remains a significant concern [6]. This is likely due to limited knowledge of the guidelines and variations in physician training [10]. ARF was selected as the focus of this study because it remains a significant and widely prevalent health concern in Saudi Arabia. This research aimed to investigate physicians' adherence to the Centor criteria when prescribing antibiotics for streptococcal pharyngitis and presents a comprehensive analysis of data collected through surveys from various healthcare settings in Jeddah. By examining this, we aimed to identify potential gaps in knowledge and practices among physicians in Jeddah, emphasizing the importance of adhering to clinical guidelines. Moreover, we hope to pinpoint areas for improvement in clinical practice and contribute to developing an evidence-based approach that enhances patient care and prevents the occurrence of ARF and its long-term complications.

## MATERIAL AND METHODS

### Sampling method

This multicenter, cross-sectional, and analytical study was conducted among physicians in primary health centers in the western region of Saudi Arabia. The main target population was physicians treating patients with sore throats. We included Saudi and non-Saudi consultants, residents, specialists in general pediatrics, family physicians, internal medicine, general physicians, and ENT departments. We excluded general surgeons, dentists, and doctors from other specialties not mentioned above.

### Study population and sampling technique

Before participating, participants were informed about the objectives of this study. The minimum required sample size was calculated using the Raosoft sample size calculator based on a 95% confidence level, a 50% expected response distribution, a 5% margin of error, and a target population of 66,014. The resulting recommended sample size was 382.

### Study tool

A structured questionnaire was developed for this study based on an extensive literature review. To ensure its reliability and validity, a pilot study was conducted involving 20 physicians to assess the clarity and relevance of the questionnaire items. In addition to the feedback from the pilot study, an expert review was conducted to refine and enhance the questionnaire’s comprehensibility and relevance. The final version of the questionnaire was designed as a brief survey to evaluate participants’ awareness and adherence to criteria for antibiotic prescription in the context of prophylaxis against acute rheumatic fever. The questionnaire consisted predominantly of direct, closed-ended questions and required less than 10 minutes to complete. Participants were contacted only at the time of the survey, with no further follow-up.

### Data analysis

Data was analyzed using the Statistical Package for the Social Sciences (SPSS) version 23.0 (SPSS Inc., Chicago, IL, USA). Data was summarized using standard deviation and mean values of age, interquartile range, and median values for Likert scores. Results were expressed in percentages for sociodemographic variables. The chi-square test was used to assess associations between categorical variables, and logistic regression analysis was performed to identify factors independently associated with adherence. A *P* value of less than 0.05 was considered statistically significant. This study was approved by the Institutional Review Board of the Faculty of Medicine at Batterjee Medical College, Jeddah. The survey was conducted anonymously, and all responses were treated as strictly confidential. Data are reported in aggregate form only, with no identifying information collected or disclosed.

## RESULTS

**Figure 1:**
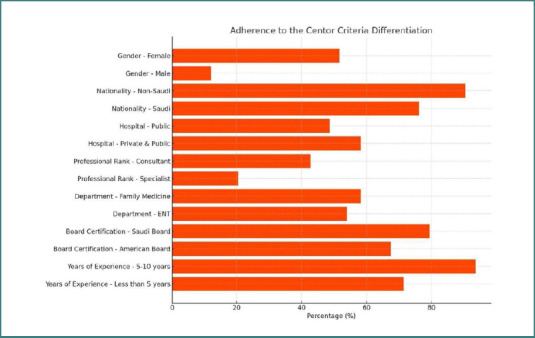
Variation in the application of Centor criteria across physician subgroups

This cross-sectional study analyzed responses from 105 physicians practicing in Jeddah, Saudi Arabia. The study evaluated their knowledge of ARF prevention and adherence to the Centor criteria for managing acute pharyngitis. The results were categorized according to the demographic characteristics of the participants, including gender, nationality, hospital affiliation, professional rank, department, board certification, and years of experience, as shown in Figure 1.

### Gender

A significant association was found between gender and the application of the Centor criteria (χ^2^ = 25.98, df = 7, *P* = 0.001). Women demonstrated higher awareness than men, with 52 women (81.3%) and 27 men (65.9%) reporting familiarity with Centor criteria. Furthermore, more women (51.6%) reported applying the Centor criteria in clinical practice compared to men (12.2%).

### Nationality

Nationality was significantly associated with adherence to ARF management guidelines (χ^2^ = 79.97, df = 16, *P* < 0.001). Non-Saudi physicians predominantly adhered to Ministry of Health (MOH) guidelines (48.1%). In contrast, Saudi physicians followed a broader range of guidelines, including the National Institute for Health and Care Excellence (NICE)/European and Infectious Diseases Society of America/Antimicrobial Stewardship Program (IDSA/ASP) protocols. Awareness of the Centor criteria was also higher among non-Saudi participants (90.4%) than Saudis (76.2%).

### Hospital

Hospital type significantly influenced the application of the Centor criteria (χ^2^ = 105.95, df = 28, *P* < 0.001). Physicians affiliated with private and public hospitals were more likely to apply the Centor criteria than those working exclusively in one sector. Furthermore, adherence to MOH guidelines was the highest among physicians in public hospitals (48.6%).

### Professional rank

Professional rank was associated with significant variations in the knowledge and application of Centor criteria. Consultants had the highest level of awareness (95.7%), followed by specialists (84.6%) and interns (78.3%) (χ^2^ = 124.1, df = 35, *P* < 0.001). Consultants were also the most likely to apply the Centor criteria in clinical practice (42.8%) (χ^2^ = 126.23, df = 49, *P* < 0.001).

### Department

Departmental affiliation showed significant differences in adherence to ARF prevention guidelines (χ^2^ = 275.04, df = 72, *P* < 0.001). Physicians in ENT and family medicine departments demonstrated the highest adherence to MOH guidelines, whereas general pediatricians and internal medicine practitioners demonstrated varied adherence to multiple international guidelines. Application of the Centor criteria was highest among family medicine physicians (58.3%).

### Board certification

**Figure 2:**
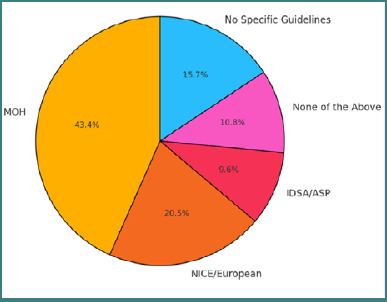
Reported use of national and international guidelines by physicians in clinical practice IDSA /ASP, Infectious Diseases Society of America / Antimicrobial Stewardship Program [11]; NICE, National Institute for Health and Care Excellence [12]; MOH, Ministry of Health [13].

Board certification was significantly associated with guideline adherence (χ^2^ = 214.95, df = 63, *P* < 0.001). Physicians holding Saudi board certification predominantly followed MOH guidelines (79.4%), while those with American or UK certifications were more likely to follow international guidelines (e.g., NICE, IDSA/ASP). Awareness of Centor criteria was also highest among those with Saudi board certification (91.2%), as shown in Figure 2.

### Years of experience

**Figure 3:**
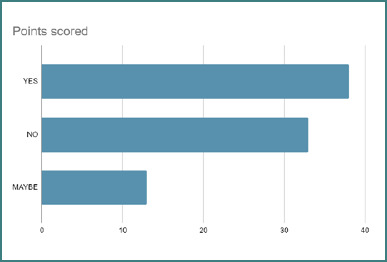
Physician self-reported application of the Centor criteria in clinical practice

Years of clinical experience were significantly associated with using the Centor criteria (χ^2^ = 155.13, df = 42, *P* < 0.001). Awareness was highest among physicians with 5–10 years of experience (93.7%) compared to those with less than 5 years (71.4%). In addition, those with more than 15 years of experience were most likely to apply the Centor criteria in practice. Nearly 40% of surveyed physicians in Jeddah reported applying the Centor criteria in their clinical practice (Figure 3).

## DISCUSSION

This cross-sectional study explored physicians' knowledge regarding the prevention of ARF and their application of the Centor criteria. A total of 105 physicians practicing in Jeddah, Saudi Arabia, participated. The study offers critical insights into the application of evidence-based guidelines in clinical practice, especially in developing regions where ARF continues to be a considerable health burden.

The results showed that almost 40% of physicians in Jeddah applied the Centor criteria (Figures 1 and 3), whereas in another study, there were significant variations in the application based on the demographic background. Female physicians demonstrated significantly greater awareness and adherence to the Centor criteria than their male counterparts. This may reflect differences in medical training or practice style. As reported by Klea *et al*., female physicians are more likely to engage in preventive care and psychosocial counseling, whereas male physicians tend to focus more on technical tasks such as history-taking and physical examination [14]. Addressing this gender gap through targeted education and training could help standardize adherence.

Nationality also had a very strong influence on guideline adherence (Figure 2). Non-Saudi physicians followed MOH guidelines more consistently, while Saudi physicians were more inclined toward international guidelines (e.g., NICE, IDSA). As highlighted by Johansen *et al*., nationality may influence the application of clinical guidelines, though it is problematic to use citizenship as a sole proxy for other background variables in medical practice [15]. Physicians may feel compelled to follow the guidelines of the country where they practice, particularly due to regulatory expectations, concerns about workplace scrutiny, or the potential impact on their residency or employment status. In our study, this dynamic was evident—non-Saudi physicians more frequently adhered to Ministry of Health (MOH) guidelines, while Saudi physicians preferred various international guidelines, such as NICE or IDSA. To address this variation, standardizing training and promoting national guidelines may enhance uniformity in clinical practice and improve adherence, especially in the context of ARF prevention, a persistent health burden in Saudi Arabia. However, another study conducted in Riyadh, Saudi Arabia, found that physicians' implementation of clinical practice guidelines was not significantly affected by their nationality. The study indicated that other factors, such as physician role, years of experience, and gender, did not significantly influence guideline adherence [16].

Another major determinant of adherence to guidelines was hospital affiliation. A study conducted in Saudi Arabia, which aimed to assess the quality of healthcare services from the patient's perspective and compare public and private hospitals (sample size: 258), found that private hospitals offered significantly higher quality care than public hospitals (t = 3.390, *P* < 0.01) [17]. This may help explain why only around 40% of physicians in our study, who focused on public health centers, applied the Centor criteria. The lower adherence in public settings could be attributed to higher patient flow and heavier workloads, which may leave physicians too fatigued to consistently apply standardized criteria. Additionally, factors such as hospital reputation and income, which are closely tied to the quality of patient care, can influence clinical behavior. In private hospitals, reputation is a strong incentive for physicians to follow guidelines closely. Interestingly, physicians working in hybrid settings (both public and private) were more likely to apply the Centor criteria than those working exclusively in either setting. This may reflect differences in case mix, patient volume, or institutional expectations. Developing these protocols at an institutional level, both in private and public healthcare, could help achieve consistent guideline outcomes and, by extension, facilitate the best possible patient care.

Professional rank significantly influenced adherence. Consultants demonstrated the highest awareness and actual use of the Centor criteria, likely reflecting their greater clinical experience and engagement in continuing medical education. In contrast, specialists and junior doctors reported lower levels of adherence. These findings denote a gap in knowledge and practice among junior physicians, which necessitates more training and mentorship for consistency in clinical decisions at all levels of practice.

Other factors contributing to the variability in guideline adherence were different departments. The physicians from the ENT and family medicine departments had better adherence to MOH guidelines, probably because of the higher prevalence of pharyngitis and ARF cases encountered in the two departments. On the contrary, pediatrics and internal medicine practitioners had marked variability in their adherence to international guidelines. This variability could be related to the patient population and diagnostic difficulties associated with each department. More importantly, standardization of training across disciplines in high-risk departments may decrease variability in the process and improve outcomes in ARF prevention.

One of the other key findings was that the association between board certification and adherence to guidelines was interrelated. Saudi board-certified physicians were more likely to adhere to MOH guidelines, while those with American or UK certification were more likely to follow international guidelines. This suggests that training and certification programs significantly influence clinical practice patterns. Efforts to develop certification curricula with national public health priorities, such as ARF prevention, may improve guideline adherence.

Finally, years of experience were very important factors in the awareness and application of the Centor criteria. Physicians with 5–10 years of experience had the highest awareness, while those with more than 15 years of experience applied the Centor criteria more consistently. This may be due to older physicians’ practical exposure over time, while younger physicians may have more current knowledge but fewer clinical encounters with ARF, which has become less common. A study by Mohammed *et al*. showed that ARF incidence declined by over 74% across two decades, limiting the younger generation’s firsthand experience [18]. However, a systematic review study by Choudhry *et al*. discussed the relationship between clinical experience and quality of health care. Surprisingly, the review of 62 studies found that over 52% indicated a decline in physician performance with increasing experience, highlighting that longer practice may not correlate with better quality of care. Only one study showed improved performance across all measured outcomes, suggesting concerns about the effectiveness of ongoing medical education [19]. This has brought out the need for continuous medical education for both early-career physicians who perhaps need more guidance in adopting evidence-based practices and experienced physicians.

Physicians' knowledge and adherence to guidelines for preventing ARF through the use of the Centor criteria and appropriate antibiotic prescription varied. While guidelines emphasize the use of clinical scoring and microbiological confirmation before prescribing antibiotics, some physicians still overprescribe due to risk aversion. The effectiveness of antibiotics in preventing ARF is well-documented. Still, based on older studies, the current low incidence of ARF in developed countries suggests that these guidelines may need updating.

### Limitations

Several limitations should be considered when interpreting the results of this study. Firstly, the sample size of 105 physicians is small and may not constitute a representative sample of physicians in Jeddah or Saudi Arabia. The low response rate and the geographic restriction to Jeddah further limit the generalizability of the findings to other regions of the country. Secondly, this study is focused only on physician self-reported practices, and it is not the direct observation of clinical behavior or outcomes, leading to differences between reported and actual adherence to guidelines. The study did not investigate the clinical outcomes of adherence to Centor criteria, such as actual ARF prevention or avoidance of antibiotic use. These are potential areas for future research to better appreciate how guideline adherence may affect patient care. Lastly, this study is limited to physicians more directly concerned with managing sore throats and streptococcal pharyngitis, whereas a wider range of healthcare providers could give a wider view into health practices concerning the prevention of ARF.

## CONCLUSION

Prevention of ARF requires a combined effort to improve the diagnosis and treatment of GAS pharyngitis. Despite ongoing efforts, ARF remains a significant public health challenge in many developing regions. The findings of this study reveal that physicians’ adherence to the Centor criteria—an essential clinical tool for diagnosing and managing streptococcal pharyngitis and, by extension, preventing ARF—varies considerably among physicians in Jeddah. Various factors included in this study were gender, nationality, professional rank, years of experience, affiliation with a particular hospital, and specialization in a certain department. Female physicians, non-Saudi practitioners, and consultants showed higher adherence rates, highlighting the effect of disparate training backgrounds and clinical experiences. These findings underscore the importance of standardized training, promotion guidelines at national levels, and continuous medical education to improve knowledge and practice gaps. Enhancing adherence to evidence-based guidelines is essential for reducing the burden of ARF in Saudi Arabia and improving the overall quality of care and health outcomes in similar high-risk populations across the developing world.

## Data Availability

Data supporting these findings are available upon request from the corresponding author.

## Personal thanks

The authors thank Dr. Mohammed ShaikhOmer for critically guiding and reviewing this article until its publication. The authors would like to thank the dedicated medical students of Batterjee Medical College, Jeddah, Saudi Arabia, Sadia Sultana, Wafa Rashid, and Juwayriyah Nawaz for their invaluable efforts in data collection.
